# An Integrated Thermopile-Based Sensor with a Chopper-Stabilized Interface Circuit for Presence Detection

**DOI:** 10.3390/s19183999

**Published:** 2019-09-16

**Authors:** Elisabetta Moisello, Michele Vaiana, Maria Eloisa Castagna, Giuseppe Bruno, Piero Malcovati, Edoardo Bonizzoni

**Affiliations:** 1 Department of Electrical, Computer and Biomedical Engineering, University of Pavia, Via Ferrata 5, 27100 Pavia, Italy; piero.malcovati@unipv.it (P.M.); edoardo.bonizzoni@unipv.it (E.B.); 2 STMicroelectronics, Str. Primosole 50, 95121 Catania, Italy; michele.vaiana@st.com (M.V.); mariaeloisa.castagna@st.com (M.E.C.); giuseppe.bruno@st.com (G.B.)

**Keywords:** chopper, CMOS, MEMS, occupancy detection, presence detection, thermal sensor, thermopile

## Abstract

This paper presents a sensor-readout circuit system suitable for presence detection. The sensor consists of a miniaturized polysilicon thermopile, realized employing MEMS micromachining by STMicroelectronics, featuring a responsivity value equal to 180 V/W, with 13 ms response time. The readout circuit is implemented in a standard 130-nm CMOS process. As the sensor output signal behaves substantially as a DC, the interface circuit employs the chopper technique in order to minimize offset and noise contributions at low frequency, achieving a measured input referred offset standard deviation equal to 1.36 μV. Measurements show that the presented system allows successfully detecting the presence of a person in a room standing at 5.5 m from the sensor. Furthermore, the correct operation of the system with moving targets, considering people either walking or running, was also demonstrated.

## 1. Introduction

Presence detection is required in a wide range of applications, from security purposes to power usage management in commercial and residential buildings. Security applications employ presence sensors to implement intruder monitoring, in private homes as well as in critical buildings, such as airports, courts, police stations, banks, government buildings, hospitals, or special laboratories (i.e., nuclear or chemical), where entrance in sensitive areas must be prevented to unauthorized subjects [1,2]. Power usage management, instead, requires presence sensors to monitor occupancy, for example in smart homes and buildings, in order to successfully control illumination, HVAC systems (i.e heating, ventilation and air conditioning) and appliances (i.e. hand dryers) in order to reduce power consumption while preserving user comfort [3,4,5,6].

Different types of systems can be employed for allowing presence detection: radio frequency identification (RFID) systems, ultrasonic sensors, carbon-dioxide (CO${}_{2}$) sensors, image-based systems, microwave wireless techniques and uncooled infrared (IR) sensors. RFID based detection systems [4] require the user to carry a radio frequency identification tag, therefore resulting unpractical. Ultrasonic sensors [7] measure the echo intensity of a transmitted signal; however they are prone to returning false positives, due to the vibrations in the surrounding environment. CO${}_{2}$ sensors [8] infer information about presence from the concentration of gas in the environment, but they are easily influenced by ambient conditions such as airflow or sensor location. Image-based systems [9,10], such as closed circuit television (CCTV) cameras, are widely used for presence detection for security purposes; however, they are rather expensive and require a significant amount of signal processing, while also giving rise to privacy issues. Microwave wireless systems [11] employ radar and Doppler radar techniques for measuring the scattering returns from the various parts of the human body during motion, including the effect of breathing and heartbeat; however, as microwave radiation propagates through many building materials, they result more suited to search and rescue applications, than to security and power management, which are the target of this work. Uncooled IR sensors, instead, are particularly well-fit for the desired applications: they feature lower cost and lower power consumption, they are small and can be easily concealed for aesthetic reasons in a smart home or if a security application requires it. Furthermore, they provide good reliability.

Pyroelectric IR (PIR) sensors [12,13,14,15,16], a particular type of uncooled thermal detectors, are the current choice for occupancy and presence detection in most buildings. However they face a severe drawback: as they only respond to the variation of incident IR radiation, they only detect motion and not stationary occupants, unless some additional expedient, such as optical and mechanical chopping, is employed. Optical chopping [15] employs an array of Fresnel lenses in order to divide the sensor field-of-view (FOV, defined as the solid angle through which the detector is sensitive to radiation) into several optically separated cones: in this way, a subject moving from one cone to the other can be detected; otherwise, as a subject moves through the FOV of the PIR only, especially if it covers a wide area, negligible changes in input IR radiation would be sensed. Mechanical chopping [3,17,18], instead, employs a shutter to modulate the radiation received by the sensor. The shutter must be moved, therefore a motor is needed, adding significantly to the power consumption the sensor intrinsically requires. Furthermore, the motor can be a source of acoustic noise. Both optical and mechanical chopping, therefore, enhance the system complexity, thus increasing its cost and reducing the advantages of employing uncooled thermal sensors.

Thermopile sensors, instead, feature the advantages of uncooled thermal detectors, while also allowing detection of stationary subjects, as they respond to incident IR radiation and not only to its variation. The drawback is that they usually have a shorter detection range with respect to PIR sensors. In this paper, however, we propose a thermopile sensor, paired with a dedicated interface circuit integrated in a separate test-chip, which achieves a detection range of 5.5 m, that is comparable with the one of PIR sensors and well satisfies the requirements for the targeted applications, i.e., intruder and occupancy detection in a room or small gateway in residential or commercial buildings, while maintaining all the advantages of uncooled thermal sensors, particularly low cost and low power consumption. The proposed thermopile sensor, featuring a 180-V/W responsivity and 13-ms response time, in fact, is realized with MEMS (Micro-Electro Mechanical Systems) micromachining by STMicroelectronics and is, therefore, fully compatible with standard CMOS processes. Furthermore, being self-powered, it does not require any biasing. The interface circuit, fabricated in a 130-nm standard CMOS process, employs chopper-stabilization technique in order to minimize the offset, achieving a measured input referred offset standard deviation equal to 1.36 μV. The proposed amplifier measured power consumption is approximately equal to 252 μV. The system performance was extensively tested, both for stationary and for moving targets, considering people either standing, walking or running at various distances from the sensor.

The paper is organized as follows: Section 2 describes the sensor-interface circuit system, Section 3 reports the measurements results and Section 4 concludes the paper.

## 2. Sensor-Interface Circuit Description

### 2.1. Thermopile Sensor

Thermopile sensors consist of *N* thermocouple elements placed in series: in this way, the sensor output signal is increased to *N* times the one of a single thermocouple element. Each thermocouple consists of two conductor materials forming electrical junctions, referred to as hot and cold junction, at different temperatures. Thanks to the Seebeck effect [19], a temperature-dependent voltage, proportional to the temperature difference between the junctions, is generated. In a micromachined thermopile sensor the hot junction features a membrane, designed to absorb IR radiation, while the cold junction corresponds to the silicon substrate and acts as a temperature reference.

**Figure 1:**
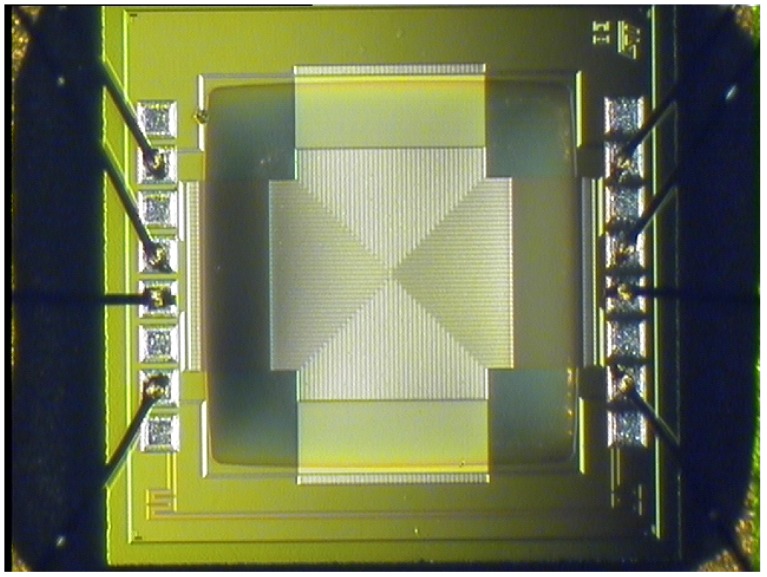
Microphotograph of the proposed micromachined thermopile sensor.

The proposed thermopile sensor [20] is fully compatible with standard CMOS processes: it employs p/n doped polysilicon as conductor materials and features a central metal plate in aluminum, embedded in a dielectric, as the absorbing membrane. The sensor is composed of 160 thermocouple elements, each measuring 250 μm in length, while the membrane area is equal to 0.64 mm${}^{2}$. The employment of polysilicon, while giving obvious advantages in terms of compatibility with standard CMOS processes, results in a larger sensor output resistance, equal to 540 kΩ, with respect to usual thermopiles. The proposed sensor, however, features an excellent responsivity, defined as the ratio between the thermopile output voltage and the incident radiant power falling on the detector, equal to 180 V/W, that is almost double with respect to typical thermopile-based sensors. The sensor time constant, equal to 13 ms, clearly satisfies the response time requirements needed by the desired applications. A microphotograph of the proposed thermopile sensor is reported in Figure 1.

**Figure 2:**
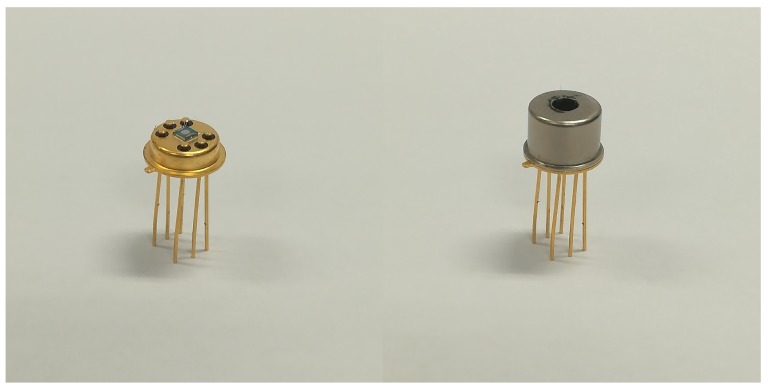
Photograph of the packaged sensor, both without and with the cap.

The sensor is packaged in a TO-5, as shown in Figure 2. In order to allow the sensor to pick up thermal radiation, the metal cap was perforated and its inner surface was covered in black opaque paint to avoid reflections. The employment of the cap was necessary in order to reduce environmental noise by limiting the FOV, which would otherwise be equal to 180${}^{\circ }$. In this first prototype no optical filter is used and, therefore, the sensor picks up radiation from the whole spectrum and not only the wavelengths corresponding to human subjects: by limiting the FOV, the impact of thermal fluctuations in the surroundings is less significant and false positive detections are avoided. The drawback is that, having a reduced FOV, the sensor covers a smaller area; however, for occupancy and presence detection applications in small gateways and rooms, such as the ones targeted in the presented work, this is not a problem. Furthermore, the proposed sensor is still a prototype and its performance could be improved by employing an appropriate optical filter.

### 2.2. Interface Circuit

**Figure 3:**
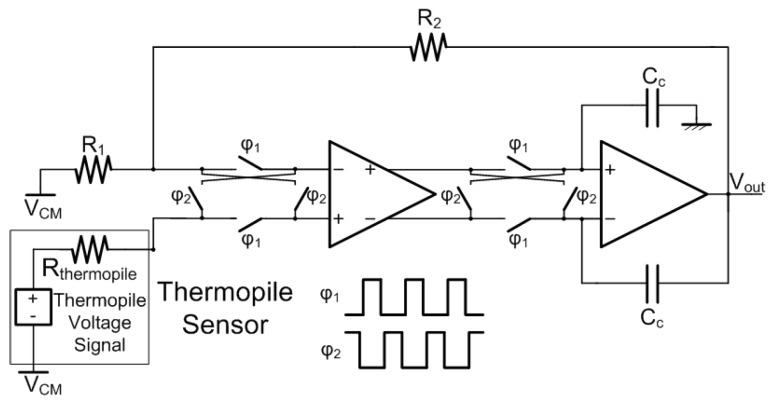
Proposed chopper amplifier architecture *©* 2019 IEEE [20].

The voltage signal produced by the sensor is in the range of few hundreds of micro-volts and behaves substantially as a DC. The interface circuit, therefore, must provide amplification while minimizing low frequency noise and offset: hence the chopper-stabilization technique [21,22] was adopted. The proposed chopper amplifier architecture [20,23] is illustrated in Figure 3. In order to achieve a more accurate control on the setting of the amplification factor, a closed-loop structure was preferred over an open-loop one. Two amplifying stages were employed to implement the required 100-dB open-loop gain. Given the thermopile sensor characteristics, namely its large output resistance and its experiencing the Perltier effect [24,25], a single-ended architecture was adopted. A closed-loop single-ended non-inverting configuration, in fact, ensures a very high, ideally infinite, input resistance, therefore preventing current flow through the thermopile and the consequent temperature variation due to the Peltier effect, which would clearly degrade the measurement. Furthermore, it allows setting the amplification factor independently from the sensor output resistance. The amplification factor in fact, chosen equal to 100, is given by $A=1+R_{2}/R_{1}$, with $R_{1}$ and $R_{2}$ equal to 1 and 99 kΩ, respectively. Capacitances $C_{c}$, equal to 220 fF, are added to ensure compensation. The supply voltage is 1.2 V, while the common-mode voltage $V_{CM}$ is set to 600 mV. CMOS switches, controlled by complementary and non-overlapping clock phases $\phi_{1}$ and $\phi_{2}$, provided through a standard disoverlap circuit, implement the modulators. The chopping frequency is 2 kHz.

**Figure 4:**
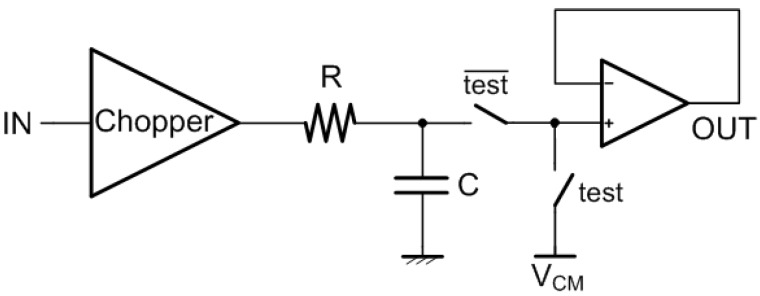
Proposed test-chip prototype architecture.

**Figure 5:**
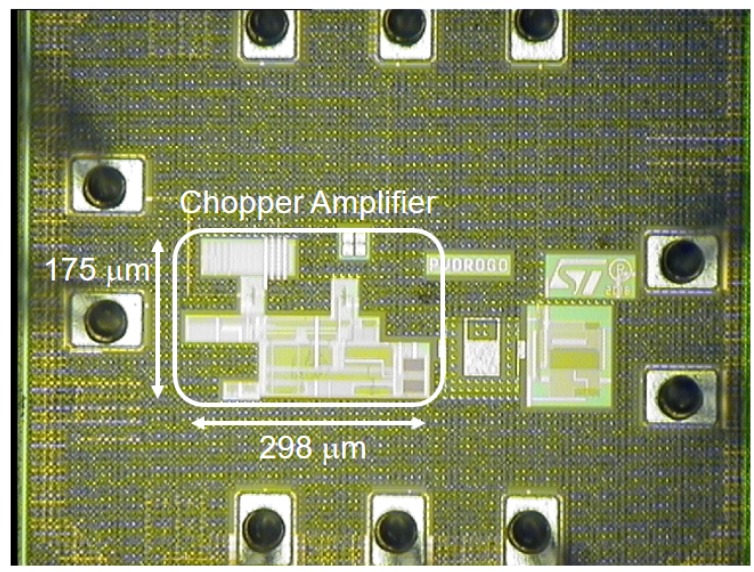
Microphotograph of the proposed test-chip prototype *©* 2019 IEEE [20].

The proposed interface circuit was implemented in a test-chip prototype, fabricated in a standard 130-nm CMOS process. A passive low-pass filter, with a cut-off frequency of 5 Hz, and a buffer were added to remove the modulated offset, moved at the chopping frequency and its odd harmonics, and to drive the output pad, respectively, as shown schematically in Figure 4. A test mode can be enabled through two CMOS switches, in order to measure the offset of the buffer and take it into account when the signal measurement is performed. Subtracting the buffer offset from the signal measurement, an input referred offset standard deviation equal to 1.36 μV was measured across 29 samples, with a 2.3-μV worst case value [20]. In the measurements reported in Section 3, however, the buffer offset was not subtracted as the interest was not on the signal absolute value, but on the signal difference between the empty room case and the one when a person was present: in the considered conditions, in fact, the buffer offset acts as a common mode signal. The test-chip prototype total power consumption is approximately 293 μW, with 14% of it required by the buffer: the advantage of low power consumption of uncooled IR sensor is therefore fully exploited. A microphotograph of the proposed interface circuit is illustrated in Figure 5. The active area is 52,150 μm${}^{2}$.

## 3. Measurements Results

### 3.1. Thermopile Sensor Responsivity Characterization

**Figure 6:**
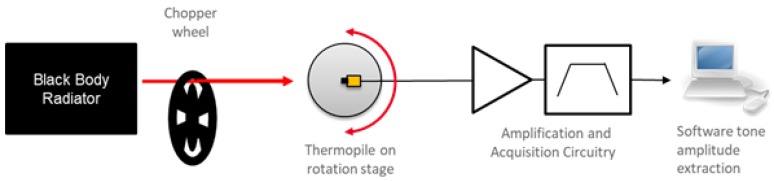
Schematic representation of the measurement setup employed for characterizing the sensor responsivity as a function of the radiation incident angle.

The thermopile responsivity as a function of the radiation incident angle was investigated considering the thermopile without the metal cap and a measurement setup as follows. The infrared radiation emitted by a black body at 300 ${}^{\circ }$C was focused on the thermopile active area. The thermopile sensor was positioned on a controlled rotation stage, in order to change the relative angle between the black body source and the sensor, thus varying the radiation incident angle. The optical excitation signal was chopped at 40 Hz, that is much higher than the thermopile cutoff frequency (12 Hz), in order to provide rejection for the low frequency noise contributed by air movements, due to air convection, instruments cooling fans and people movements. Figure 6 illustrates the experimental setup schematic.

**Figure 7:**
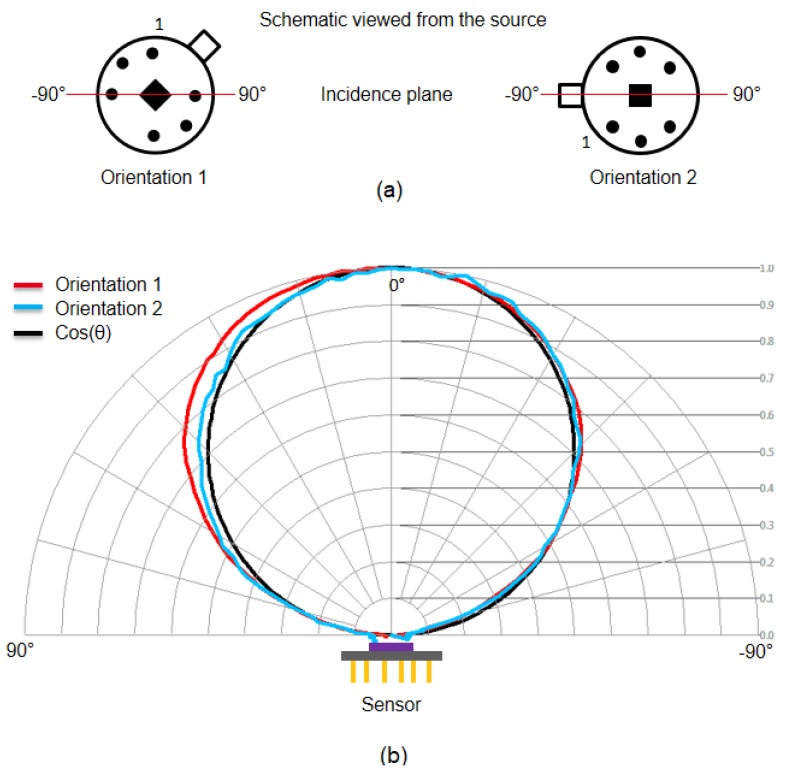
(**a**) Considered orientations of the thermopile sensor; (**b**) photo-metric diagram.

The characterization was performed considering two different orientations of the device under test as reported in Figure 7a. The measured response for the different incident angles θ, normalized to the maximum measured value, considering both orientations, is reported in the photo-metric diagram of Figure 7b. The obtained photo-metric diagram can be well fitted with cos(θ), as shown in Figure 7b: the thermopile surface can be in fact considered a lambertian surface, since no filters or lenses are integrated on the thermopile active surface [26]. The response can be considered independent from the orientation.

### 3.2. System Performance at Different Ambient Temperatures

**Figure 8:**
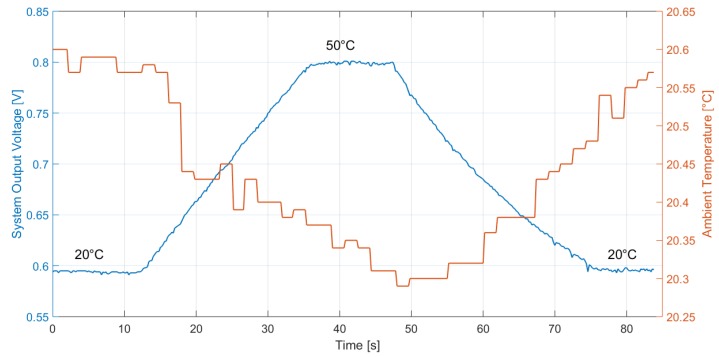
Measured thermopile sensor-interface circuit system output with the black body at 10-cm distance and the ambient temperature at 20 ${}^{\circ }$C. The black body temperature is varied in ramp fashion from 20 ${}^{\circ }$C to 50 ${}^{\circ }$C. The buffer offset was not subtracted from the output signal.

**Figure 9:**
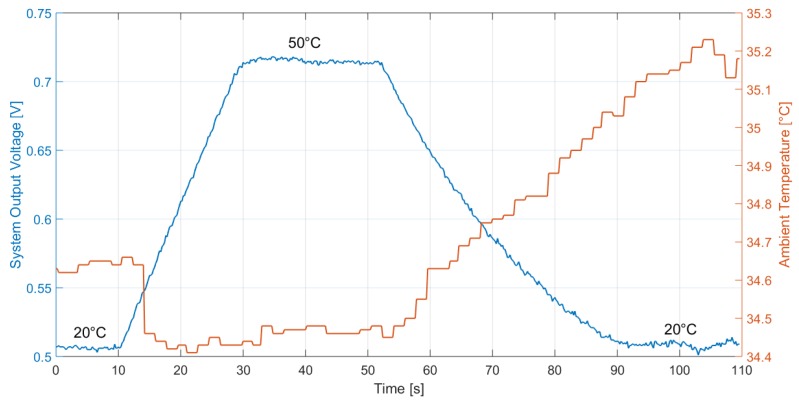
Measured thermopile sensor-interface circuit system output with the black body at 10-cm distance and the ambient temperature at 35 ${}^{\circ }$C. The black body temperature is varied in ramp fashion from 20 ${}^{\circ }$C to 50 ${}^{\circ }$C. The buffer offset was not subtracted from the output signal.

The proposed thermopile sensor and interface circuit, integrated in two separate test chips, were tested together as a system to perform presence detection for occupancy and intruder monitoring applications. In order to shield the sensor from air movements and environmental noise, a metal cap, such as the one illustrated in Section 2.1, was employed. In order to verify the correct operation for different room temperatures, the system was tested in a climatic chamber, employing a black body radiator (SR-800R 4D/A model by CI Systems [27]) as target object, placed at 10 cm from the sensor. The black body temperature was varied in ramp fashion from 20 ${}^{\circ }$C to 50 ${}^{\circ }$C and back, while monitoring the climatic chamber temperature. The system output signal, while applying a common mode voltage equal to 600 mV, was measured in the case of 20 ${}^{\circ }$C and 35 ${}^{\circ }$C ambient temperature in the climatic chamber: the results are illustrated in Figure 8 and Figure 9, respectively. The system output voltage acquisition was performed through a Keithley 2001 multimeter, coupled with a LabVIEW program, at a 5-Hz rate.

The system sensitivity, without removing the buffer offset, can be estimated as

(1) $$S=\frac{Output\;Signal_{\left(T=50^{\circ }C\right)}-Output\;Signal_{\left(T=20^{\circ }C\right)}}{{\left(50-20\right)}^{\circ }C}$$

where *T* is the target object temperature. It results in S = 6.90 mV/${}^{\circ }$C and S = 7.03 mV/${}^{\circ }$C for 20 ${}^{\circ }$C and 35 ${}^{\circ }$C, respectively: the system performance, therefore, is substantially independent of ambient temperature, while considering a typical range of room temperature values.

### 3.3. Presence Detection of Stationary Subjects

**Figure 10:**
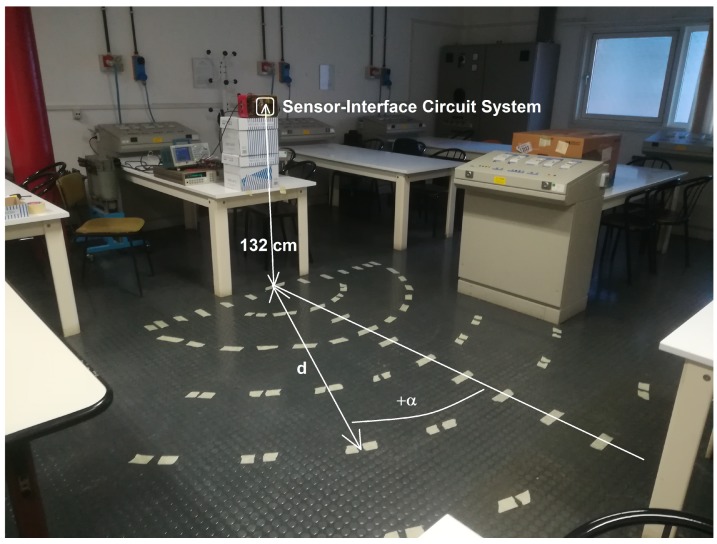
Room used for presence detection testing of a stationary subject. The signs on the floor identify the various considered person’s locations.

**Figure 11:**
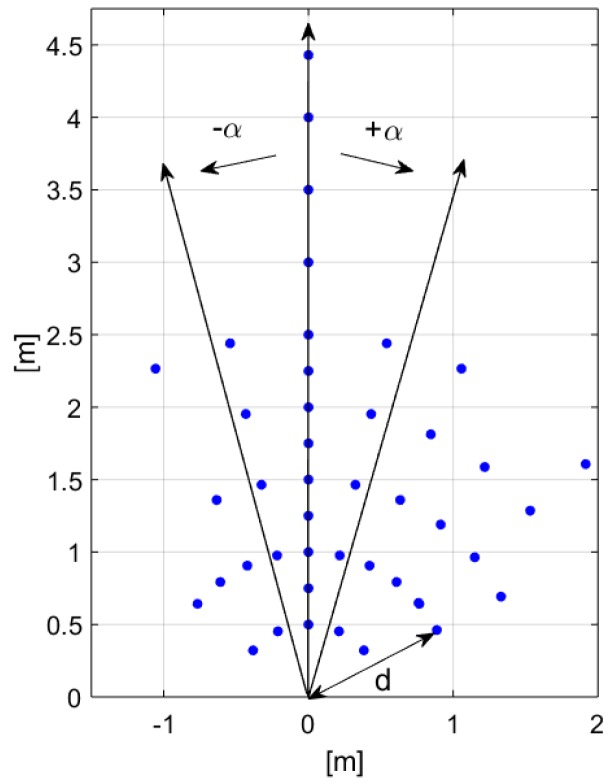
Person’s locations in the room considered for presence detection testing of a stationary subject.

The sensor-interface circuit system was tested for presence detection of stationary subjects, considering a person standing in a room at various distances *d* and angles α from the sensor. The sensor, with the metal cap, and the interface circuit, inserted in their respective boards and connected together, were placed at a 132-cm height from the ground, facing the room. Several different locations, identified by *d* and α and signaled on the floor by means of white tape, were considered, as illustrated in Figure 10. The chosen locations are reported in Figure 11.

**Figure 12:**
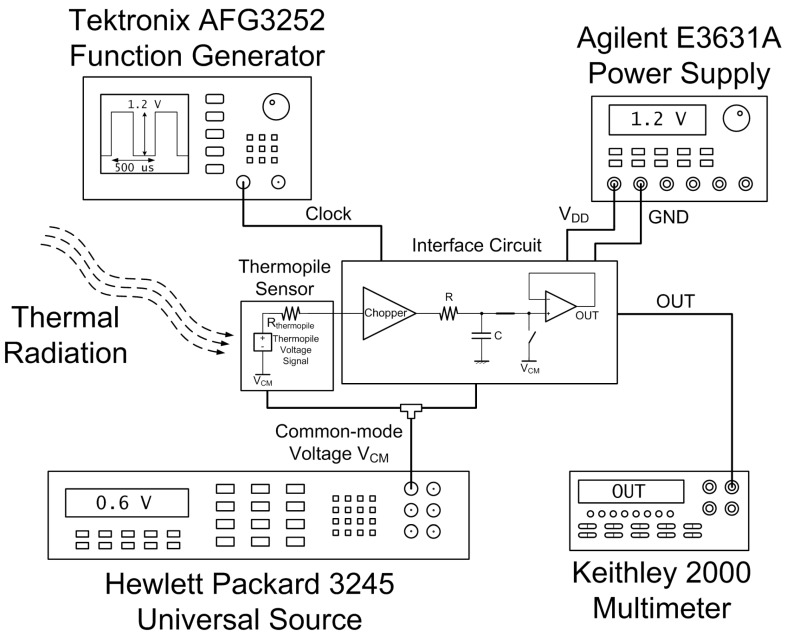
Schematic view of the measurement setup.

The supply voltage for the interface circuit, equal to 1.2 V, was provided through an Agilent E3631A power supply, while a Hewlett Packard 3245 universal source supplied the common-mode voltage, equal to 600 mV. The circuit bias current was regulated through a resistor on-board and set equal to approximately 70 μA. A Tektronix AFG3252 function generator supplied the 2-kHz 0–1.2-V square wave clock signal for the chopper. A Keithley 2000 multimeter was employed to measure the system output voltage. The schematic view of the measurement setup is illustrated in Figure 12.

The measurements were performed at 22 ${}^{\circ }$C room temperature, considering a 1.75-m average-build man (wearing trousers, a shirt and a sweater) as the stationary subject. For each identified location, 100 output acquisitions at 1.25 Hz (i.e., the multimeter *slow rate*) were performed considering both the case with the person standing and the empty room case. The measurement results for each case were stored in the buffer of the multimeter, which then returned the average and standard deviation value. The difference between the average in the occupied room case and in the empty room case was then considered as the output signal of interest. The results are reported in Table A1 in Appendix A.

**Figure 13:**
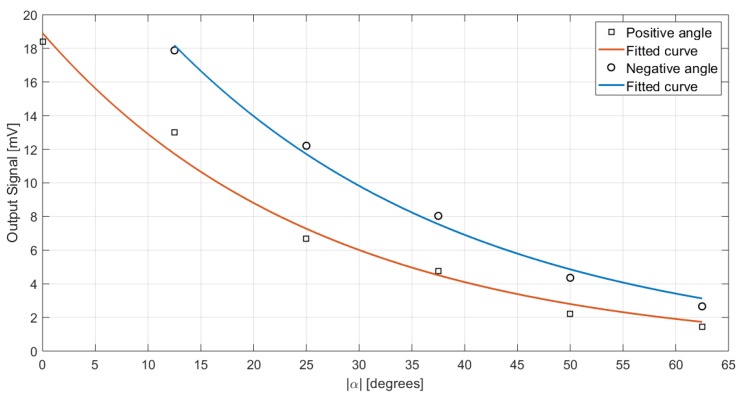
Signal in the case of a person standing at d = 1 m from the sensor for different values of α.

The output signal in the case of a subject at a fixed 1-m distance and different angles from the sensor is reported in Figure 13. The fitted curves for positive and negative values of α share the same shape and are clearly superimposable, therefore the measurements are symmetrical. However, as the curves are not coincident and differ in average for a −13${}^{\circ }$ shift between the positive and the negative angle curves, they are not symmetrical across the line previously identified as corresponding to 0${}^{\circ }$: this is due to the fact that the chosen 0${}^{\circ }$-line is not perpendicular to the sensor surface because of alignment inaccuracies during the setup.

In order to have the actual sensor’s normal line as reference, the following angle correction was performed:

(2) $$\alpha_{corrected}=\alpha -\theta$$

where

(3) $$\theta =\frac{\mathrm{Average}\;\mathrm{difference}\;\mathrm{between}\;\mathrm{positive}\;\mathrm{angle}\;\mathrm{curve}\;\mathrm{and}\;\mathrm{negative}\;\mathrm{angle}\;\mathrm{curve}}{2}=\frac{-13^{\circ }}{2}=-6.5^{\circ }$$

**Figure 14:**
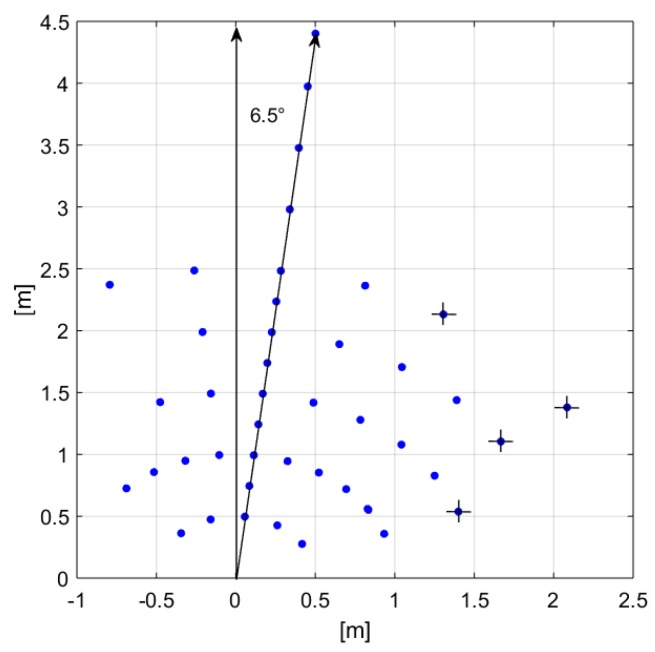
Person’s locations in the room considered for presence detection testing of stationary subjects, applying the angle correction.

Figure 14 illustrates the standing subject locations referred to the sensor’s normal. The points with a cross indicate that the system was not able to detect the subject presence in that location. Presence detction was considered achieved if a positive output signal was found considering a ±2σ variation, where σ is the worst case standard deviation between the empty and the occupied room value reported in Table A1. The measured sensor FOV is equal approximately to 120${}^{\circ }$ at 1-m distance.

**Figure 15:**
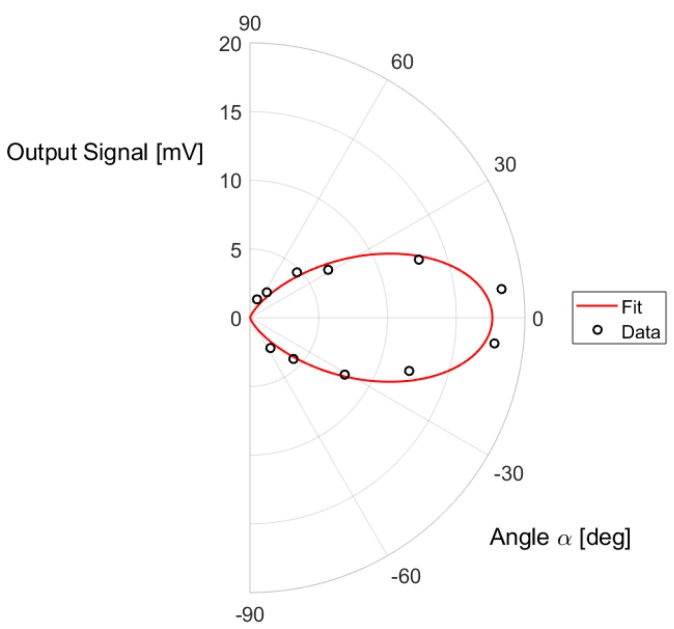
Graphical comparison between the derived fit and the measured data in the case of 1-m fixed distance.

Taking into account the angle correction, the dependency on both the angle, $\alpha_{corrected}$, and the distance, *d*, of the subject from the sensor was investigated in order to derive the best fit for the measurement results. The Matlab Curve Fitting Tool was employed in the process. Considering the results for 1-m fixed distance and different angle values, the identified fit function is

(4) $$\mathrm{Output}\;\mathrm{Signal}=c_{a1}\;{\left(cos\;\alpha_{corrected}\right)}^{c_{a2}}$$

where $c_{a1}$ and $c_{a2}$ are equal to 17.63 and 4.766, respectively, considering 95% confidence bounds. The fit yields a correlation with R-squared equal to 0.9621 and a root mean square error (RMSE) equal to 1.262. A graphical representation of the measured data and the derived fit is reported in Figure 15. The value of $c_{a2}$ well matches with the expected value from the theory: in the case of extended lambertian sources (e.g., the human body) parallel to the detector, in fact, the radiant intensity is proportional to ${\left(cos\;\alpha_{corrected}\right)}^{4}$ [28].

**Figure 16:**
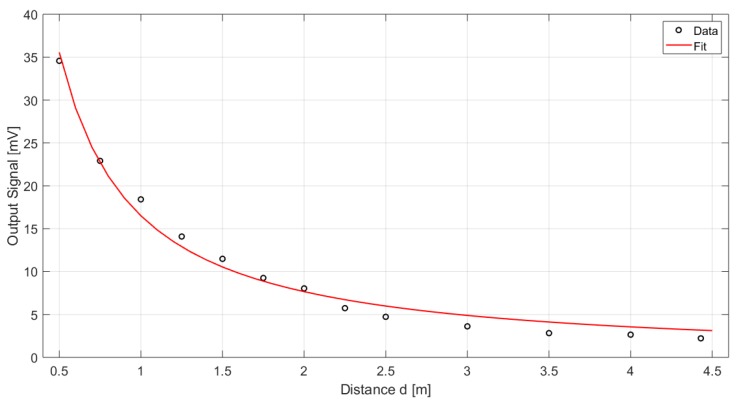
Graphical comparison between the derived fit and the measured data in the case of 6.5${}^{\circ }$ fixed angle.

Considering instead the measurements results for a 6.5${}^{\circ }$ fixed angle at various distances, the derived fit function is

(5) $$\mathrm{Output}\;\mathrm{Signal}=\frac{c_{d1}}{d^{c_{d2}}}$$

where $c_{d1}$ and $c_{d2}$ are equal to 16.51 and 1.107, respectively, considering 95% confidence bounds. The R-squared coefficient and the RMSE are equal to 0.9866 and 1.163, respectively. Figure 16 illustrates a graphical representation of the measured data and the derived fit.

**Figure 17:**
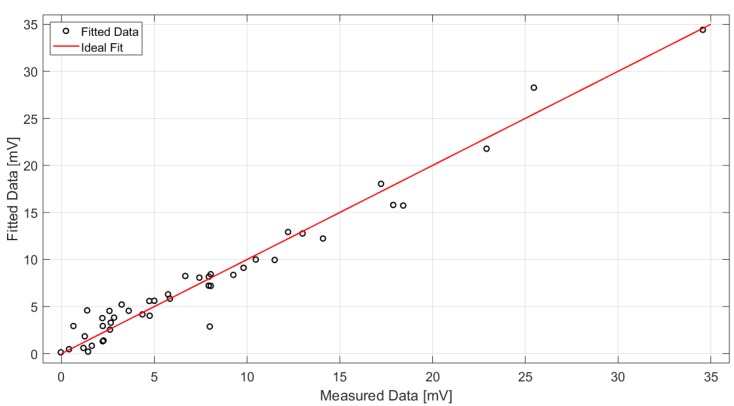
Graphical comparison between the derived fit and the ideal fit.

The derived curves well fit the measured data in the fixed-distance/variable-angle and fixed-angle/variable-distance cases; the fit shape in the case of both variable angle and variable distance, therefore, was chosen equal to the product between the two curves shapes. The derived fit function is

(6) $$\mathrm{Output}\;\mathrm{Signal}=c_{1}\;{\left(cos\;\alpha_{corrected}\right)}^{c_{2}}\;\frac{1}{d^{c_{3}}}$$

where $c_{1}$ = 16.17, $c_{2}$ = 4.222 and $c_{3}$ = 1.129, with 95% confidence bounds. The fit yields 0.9599-R-squared and 1.52-RMSE: it is therefore a good approximation for the measurements results, as shown in Figure 17.

**Figure 18:**
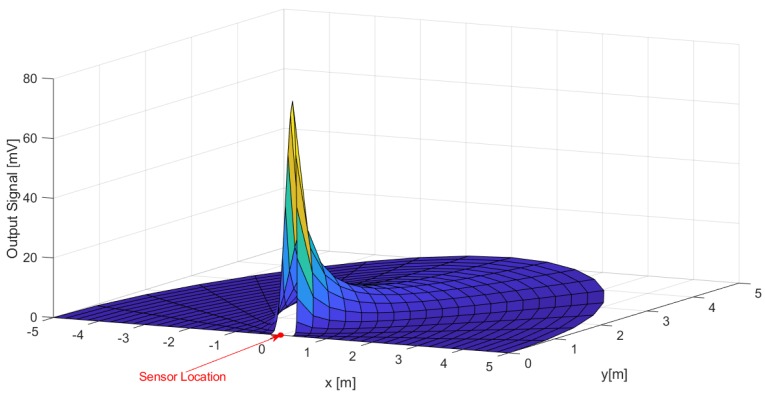
3-D graphical representation of the derived fit in cartesian coordinates.

The 3-D representation of the derived fit as a function of the stationary subject location, expressed in a cartesian coordinate system where the y-axis is the normal to the sensor surface, is illustrated in Figure 18.

**Figure 19:**
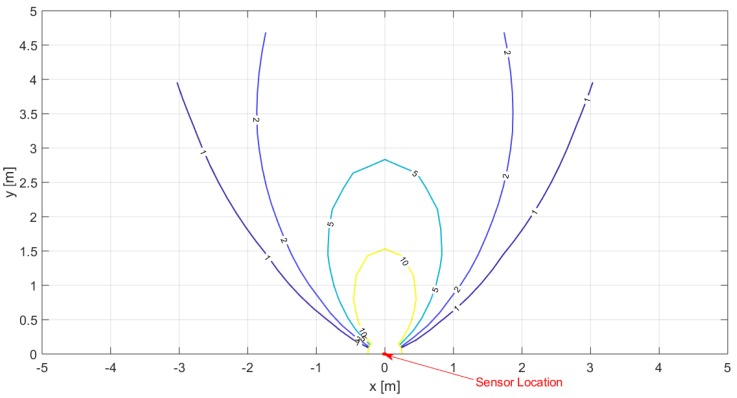
2-D representation of the sensor detection range, obtained from the derived fit. The reported lines are the isolines corresponding to an output signal equal to 10 mV, 5 mV, 2 mV and 1 mV.

A 2-D representation of the sensor detection range, derived from the obtained fit, with isolines corresponding to 10, 5, 2 and 1 mV output signal acting as delimiters, is reported in Figure 19. The derived FOV well satisfies the specifications for the targeted applications, which require a short-distance (few meters) detection capability. The maximum measured detection distance in the considered setup was 4.43 m. The limit, however, was imposed by the room size: therefore, in order to verify fully the sensor detection capability, the setup was moved to a larger room and the measurements for a 0${}^{\circ }$-line were repeated, considering a 1.65-m average-build woman (wearing jeans and a t-shirt) as the stationary subject. The measurements results are reported in Table A2 in Appendix A.

Considering a ±2σ variation as stated before, a positive output signal was found for distances up to 5.5 m. Employing the Matlab Curve Fitting Tool, adopting the same fit function as for the other measurements set, the fit for the measured output signal as a function of the distance was derived

(7) $$\mathrm{Output}\;\mathrm{Signal}=\frac{c_{d1}}{d^{c_{d2}}}$$

where $c_{d1}$ = 25.71 and $c_{d2}$ = 1.07, with 95% confidence bounds. R-squared and the RMSE are equal to 0.9627 and 2.995, respectively.

The correlation coefficient, R-squared, between the fit functions of the two measurements sets in the fixed-angle/variable-distance case is 0.9999: the measurements, therefore, are clearly repeatable, even when varying the room setting and the stationary subject.

### 3.4. Presence Detection of Moving Subjects

**Figure 20:**
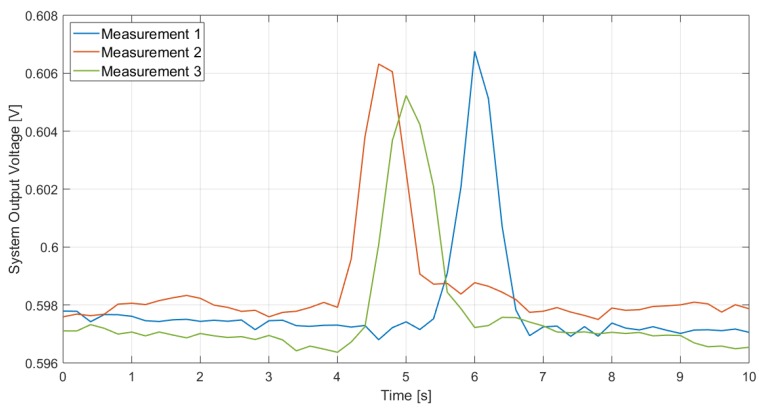
Measuremets results in the case of a subject walking at 1 m in front of the sensor. The sensor is located at a 132-cm height from the ground.

The system performance in the presence of moving subjects was also tested. The same room setup illustrated in Figure 10 was adopted and a 1.70-m average-build woman (wearing trousers and a t-shirt) was considered as moving subject. The subject moved in a straight line, approximately perpendicular to the sensor’s normal, at different distances from the sensor. The measurements were performed acquiring the system output voltage through a Keithley 2000 multimeter, paired with a LabVIEW program. The chosen acquisition rate was 5 Hz when the subject was walking and 10 Hz when the subject was running. A common-mode voltage equal to 600 mV was supplied through the universal source. The ambient temperature was roughly equal to 26.5 ${}^{\circ }$C. Figure 20 illustrates the measurements results when the subject is walking at 1 m from the sensor, which is located at a 132-cm height from the ground. The system performance is validated as a peak, corresponding to when the subject moves within the sensor FOV, is clearly distinguishable. Different measurements were performed and repeatability was verified.

**Figure 21:**
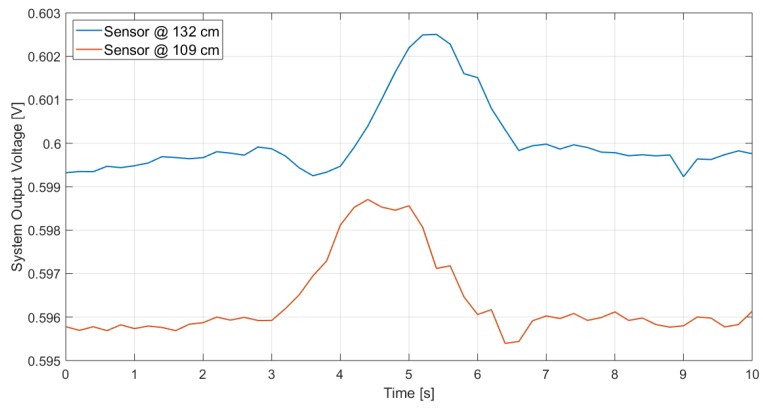
Measurements results in the case of a subject walking at 2.5 m in front of the sensor, for different sensor heights.

**Figure 22:**
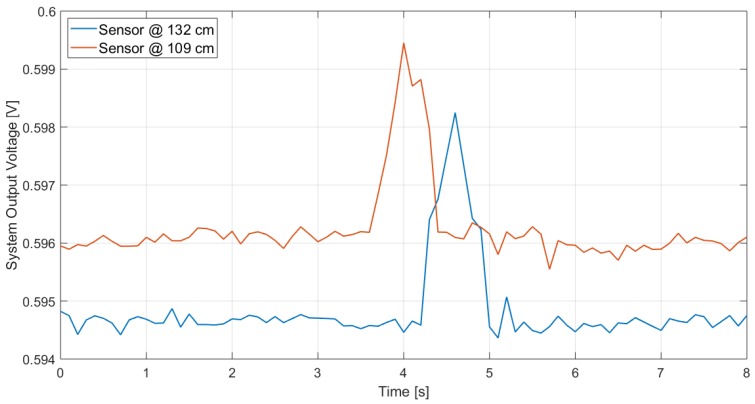
Measurements results in the case of a subject running at 2.5 m in front of the sensor, for different sensor heights.

Furthermore, the system performance was investigated considering a larger distance, different sensor’s height from the ground and different subject’s speed (walking or running). The results are reported in Figure 21 and Figure 22, which illustrate the measurement results when the subject is, respectively, walking and running at 2.5 m from the sensor, when that is located at 132-cm and 109-cm height from the ground: the peak, which detects the subject presence, is clearly visible in all cases. As expected, the peak lasts longer when the subject is walking as in that case the subject, being slower, remains longer within the sensor FOV. Furthermore, considering the same subject speed, the peak at 2.5 m lasts longer than the one for 1 m: this is due to the fact that the area covered by the sensor at a given distance corresponds to the section of a solid angle and, therefore, the one at 2.5 m is larger than the one at 1 m; hence, the subject remains in the sensor FOV longer.

It is to be noted that the system output voltage when no subject is detected varies of a few mV from one measurement to the other: this is simply due to the fact that the measurements were performed at different moments and, therefore, were subject to temperature variations in both the ambient and the sensor, that, however small, given the sensor excellent responsivity and therefore sensitivity, would result in a voltage difference. However, this is not an issue: the peak, in fact, is the one that enables presence detection; we are, therefore, interested in the relative variation of the system output voltage, not in its absolute value.

## 4. Conclusions

This paper presented a sensor-readout circuit system suitable for presence detection for security applications as well as occupancy monitoring for power usage management in commercial and residential buildings. The sensor, consisting of a miniaturized micromachined polysilicon thermopile with 180-V/W responsivity and 13-ms response time, features low power consumption, being self-biased, and low cost, as it is compatible with standard CMOS processes. The interface circuit, whose design was tailored on the sensor’s specific characteristics, was fabricated in a standard 130-nm CMOS process and features a 1.36-μV measured input referred offset standard deviation across 29 samples. The sensor and the interface circuit, integrated in two separate chips in this first prototype realization, were extensively tested together as a system to perform presence detection. The system performance, considering both moving and stationary subjects, was verified for different subjects, different room temperatures, different subject-sensor distances and different sensor’s heights from the ground: the obtained results can, therefore, be generalized for any average-build man or woman in an indoor space at typical room temperature values. Considering a stationary subject, measurements show that the presented system is able to successfully detect the subject presence at 5.5-m distance from the sensor, that is comparable with the performance achieved by PIR sensors, usually employed for the targeted applications. Unlike PIR sensors, however, this system, thanks to the thermopile principle of operation, is intrinsically able to detect both moving and stationary subjects, without the need of optical or mechanical chopping.

## Appendix A

*d* and α identify the subject location as illustrated in Figure 10 and Figure 11. For each identified subject location, 100 system output acquisitions at 1.25 Hz were performed with a multimeter, considering both the case with the person standing (occupied room) and the empty room case. The measurements results for each case were stored in the buffer of the multimeter, which then returned the average and standard deviation value. The difference between the average in the occupied room case and in the empty room case is considered as the output signal of interest.

**Table A1:** Measurements Results for Different Person Locations.

d [m]	α [degrees]	Output Signal [mV]	Output Std [mV] (Empty Room)	Output Std [mV] (Occupied Room)
0.5	0	34.57	0.414	1.419
0.75	0	22.91	0.414	0.594
1	0	18.42	0.441	1.036
1.25	0	14.09	0.441	0.459
1.5	0	11.49	0.562	0.591
1.75	0	9.26	0.562	0.546
2	0	8.04	0.813	0.691
2.25	0	5.74	0.813	0.573
2.5	0	4.73	0.724	0.521
3	0	3.62	0.433	0.38
3.5	0	2.83	0.504	0.324
4	0	2.65	0.504	0.611
4.43	0	2.22	0.4	0.553
1	12.5	12.99	0.561	0.57
1.5	12.5	7.43	0.561	0.45
2	12.5	5.84	0.692	0.464
2.5	12.5	2.58	0.692	0.763
0.5	25	17.22	0.544	0.552
1	25	6.67	0.744	0.439
1.5	25	3.24	0.744	0.536
2	25	2.2	0.52	0.739
2.5	25	0.64	0.52	0.617
1	37.5	4.75	0.564	0.763
1.5	37.5	2.61	0.586	0.399
2	37.5	1.25	0.586	0.39
2.5	37.5	1.38	0.544	0.363
0.5	50	7.99	0.589	0.7
1	50	2.22	0.589	0.789
1.5	50	1.63	0.769	0.565
2	50	1.18	0.564	0.671
2.5	50	0.4	0.755	0.496
1	62.5	1.43	0.589	0.709
1.5	62.5	−0.04	0.589	0.664
1	−12.5	17.88	0.575	1.062
1.5	−12.5	10.47	0.575	0.923
2	−12.5	7.93	0.7	0.891
2.5	−12.5	5	0.684	0.753
0.5	−25	25.46	0.755	0.995
1	−25	12.21	0.684	0.74
1.5	−25	7.94	0.684	0.992
1	−37.5	8.04	0.755	0.833
0.5	−50	9.81	0.755	0.695
1	−50	4.36	0.755	0.523
1	−62.5	2.66	0.755	0.455

**Table A2:** Measurements Results for Different Person Locations.

d [m]	α [degrees]	Output Signal [mV]	Output Std [mV] (Empty Room)	Output Std [mV] (Occupied Room)
0.5	0	49.52	1.055	0.999
0.75	0	40.28	1.055	0.981
1	0	29.12	0.878	0.67
1.25	0	23.17	0.878	1.027
1.5	0	18.52	1.041	1.091
1.75	0	14.78	1.041	1.559
2	0	9.23	1.231	1.529
2.5	0	7.36	1.231	1.420
3	0	5.19	1.413	1.670
3.5	0	5.80	1.413	1.290
4	0	3.34	1.122	1.381
4.5	0	2.16	1.122	1.743
5	0	3.57	0.987	1.332
5.5	0	3.97	0.987	1.295
6.5	0	1.08	1.225	2.168
